# Excitatory Dendritic Mitochondrial Calcium Toxicity: Implications for Parkinson’s and Other Neurodegenerative Diseases

**DOI:** 10.3389/fnins.2018.00523

**Published:** 2018-08-02

**Authors:** Manish Verma, Zachary Wills, Charleen T. Chu

**Affiliations:** ^1^Department of Pathology, School of Medicine, University of Pittsburgh, Pittsburgh, PA, United States; ^2^Department of Neurobiology, School of Medicine, University of Pittsburgh, Pittsburgh, PA, United States; ^3^Department of Ophthalmology, School of Medicine, University of Pittsburgh, Pittsburgh, PA, United States; ^4^Pittsburgh Institute for Neurodegenerative Diseases, School of Medicine, University of Pittsburgh, Pittsburgh, PA, United States; ^5^McGowan Institute for Regenerative Medicine, School of Medicine, University of Pittsburgh, Pittsburgh, PA, United States; ^6^Center for Protein Conformational Diseases, School of Medicine, University of Pittsburgh, Pittsburgh, PA, United States; ^7^Center for Neuroscience, School of Medicine, University of Pittsburgh, Pittsburgh, PA, United States

**Keywords:** mitochondrial calcium uniporter, PINK1, LRRK2, calcium overload, Parkinson Disease/Lewy body dementia, Alzheimer Disease, FTD-ALS, dendrite degeneration

## Abstract

Dysregulation of calcium homeostasis has been linked to multiple neurological diseases. In addition to excitotoxic neuronal cell death observed following stroke, a growing number of studies implicate excess excitatory neuronal activity in chronic neurodegenerative diseases. Mitochondria function to rapidly sequester large influxes of cytosolic calcium through the activity of the mitochondrial calcium uniporter (MCU) complex, followed by more gradual release via calcium antiporters, such as NCLX. Increased cytosolic calcium levels almost invariably result in increased mitochondrial calcium uptake. While this response may augment mitochondrial respiration, limiting classic excitotoxic injury in the short term, recent studies employing live calcium imaging and molecular manipulation of calcium transporter activities suggest that mitochondrial calcium overload plays a key role in Parkinson’s disease (PD), Alzheimer’s disease (AD), amyotrophic lateral sclerosis (ALS), and related dementias [PD with dementia (PDD), dementia with Lewy bodies (DLB), and frontotemporal dementia (FTD)]. Herein, we review the literature on increased excitatory input, mitochondrial calcium dysregulation, and the transcriptional or post-translational regulation of mitochondrial calcium transport proteins, with an emphasis on the PD-linked kinases *LRRK2* and *PINK1.* The impact on pathological dendrite remodeling and neuroprotective effects of manipulating MCU, NCLX, and LETM1 are reviewed. We propose that shortening and simplification of the dendritic arbor observed in neurodegenerative diseases occur through a process of excitatory mitochondrial toxicity (EMT), which triggers mitophagy and perisynaptic mitochondrial depletion, mechanisms that are distinct from classic excitotoxicity.

## Introduction

Neuronal function is dependent upon the formation, maintenance, and activity-regulated remodeling of multiple synaptic contacts supported by extensive axo-dendritic arborization. The primary excitatory neurotransmitter is glutamate. Glutamate binds to calcium-permeable ionotropic receptors that are also activated by *N*-methyl-D-aspartate (NMDA) or α-amino-3-hydroxy-5-methyl-4-isoxazolepropionate (AMPA). These NMDA receptors (NMDAR) and AMPA receptors (AMPAR) are present predominantly in dendritic spines, but also exist in perisynaptic regions. NMDA receptors are composed of NR1 and NR2 subunits; the four NR2A-NR2D subunits confer different kinetic properties, channel open probabilities, ion conductance, and effects on synaptic plasticity. Excitatory synaptic activity engages NMDAR subsets that contain the NR2A subunit, resulting in activation of Akt, ERK1/2, and CREB (Lau and Zukin, 2007). In addition to ligand-gated channels, L-type voltage gated calcium channels are also involved in activating ERK1/2 and CREB to regulate activity-dependent transcription (West et al., 2002). Transient activation of these signaling pathways is implicated in neuronal survival as well as in synaptic plasticity.

Due to its essential role in signaling both pre-synaptic and post-synaptic processes, as well as cellular processes of differentiation, cell death, vesicular transport, and cytoplasmic motility, calcium undergoes exquisitely precise regulation in neurons that allow simultaneous engagement of multiple spatially separated calcium-dependent processes. Dendritic spines function as physical compartments that isolate and concentrate calcium signals arising from synaptic activity (Koch et al., 1992). Following depolarization or ligand-stimulated calcium uptake, calcium signal recovery is mediated by channel inactivation, plasma membrane sodium-calcium exchangers (NCX) that extrude calcium, and sequestration of calcium into mitochondria, endoplasmic reticulum, and other intracellular stores. The mitochondrion plays a key role in rapid, post-stimulatory calcium recovery by taking up massive amounts of calcium into its matrix (White and Reynolds, 1997), while also fueling ATP-dependent pumps on other membranes (Budd and Nicholls, 1996).

While increased excitatory stimulation has been extensively studied in the context of acute neuronal injury and cell death, it has become clear in recent years that increased neuronal calcium handling may also play a pathogenic role in chronic neurodegenerative diseases. Shortening and simplification of the dendritic arbor and spine loss, often accompanied by loss of dendritic mitochondria (Cherra et al., 2013; Dagda et al., 2014) are observed in post-mortem studies of Alzheimer’s disease (AD), Parkinson’s disease (PD), and amyotrophic lateral sclerosis (ALS) (Hammer et al., 1979; Patt et al., 1991; Baloyannis et al., 2004; Stephens et al., 2005) or their experimental models (MacLeod et al., 2006; Wu et al., 2010; Dagda et al., 2014; Fogarty et al., 2016). Although inhibiting calcium uptake from the extracellular space is frequently neuroprotective (Ilijic et al., 2011; Cherra et al., 2013; Esposito et al., 2013; Plowey et al., 2014), the mechanism(s) by which sublethal increases in cytosolic calcium fluxes trigger dendritic retraction have been unclear. A series of recent studies discussed below implicate increased mitochondrial calcium stress as a key factor by which increased excitatory neuronal activity triggers mitochondrial depletion from and retraction of dendritic structures. Moreover, several mitochondrial calcium transporters are regulated by genes mutated in familial PD, causing functional changes that increase susceptibility to this neurodegenerative mechanism, which we have termed excitatory mitochondrial toxicity (EMT). Genetic and aging- or disease-related signaling alterations may also predispose to EMT in sporadic PD, AD, and the ALS-frontotemporal dementia (FTD) spectrum.

## A Brief Summary of Excitotoxicity

Over the past 40–50 years, it has become well recognized that excessive glutamatergic neurotransmission leads to neuronal cell death, which was first described by Olney (1971). While this has been studied most extensively in the context of brain ischemia from stroke or trauma, excitotoxic cell death has also been implicated in epilepsy and to a lesser extent in AD (Tannenberg et al., 2004), PD (Caudle and Zhang, 2009), and ALS (Shi et al., 2010).

**FIGURE 1:**
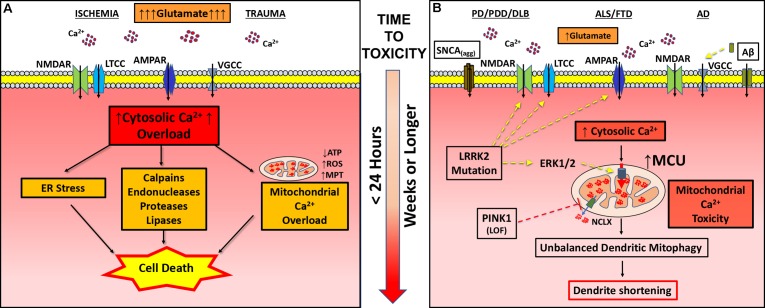
Pathogenic mechanisms in excitotoxicity and dendritic EMT. **(A)** In classic excitotoxicity an insult, such as ischemia or trauma, causes increased release of excitatory neurotransmitters (glutamate) leading to post-synaptic uptake of calcium through channels (AMPAR, α-amino-hydroxy-5-methyl-4-isoxazolepropionic acid receptor; LTCC, L-type calcium channel; NMDAR, *N*-methyl-D-aspartate receptor; VGCC, voltage gated calcium channel). Falling ATP levels impair calcium pumps, contributing to cytosolic calcium overload. Sustained intracellular calcium elevation activates a variety of degradative enzymes (calpains, endonucleases, proteases, and lipases), ER stress and mitochondrial permeability transition (MPT), leading to cell death within hours of initiation of excitotoxicity. **(B)** In neurodegenerative diseases (PD/PDD/DLB, ALS/FTD, and AD), various changes including enhanced glutamate neurotransmission contribute to increased cytosolic calcium flux. In PD/PDD/DLB, aggregated α-synuclein (SNCA_agg_) can insert into the membrane forming calcium permeable pores. Mutations in the LRRK2 gene contribute to more frequent excitatory post-synaptic potentials and increased calcium influx via NMDAR, AMPAR, and/or LTCC. In AD, soluble amyloid beta (Aβ) can either directly stimulate VGCC or aggregates to form calcium permeable pores. Chronically elevated calcium transients, while insufficient in magnitude to trigger calcium-dependent cell death, results in greater calcium uptake by mitochondria. In addition to elevating cytosolic calcium, mutations in LRRK2 (R1441C/G2019S) transcriptionally upregulate MCU through activation of ERK1/2. On the other hand, loss of function (LOF) mutations in PINK1 result in impaired activation of NCLX-mediated calcium efflux from the mitochondria. Dysregulation of mitochondrial calcium handling, whether driven by increased excitatory cytosolic calcium uptake or changes in the function of mitochondrial calcium transporters, results in mitochondrial injury sufficient to trigger mitophagy. Depletion of dendritic mitochondria precedes subsequent shortening and simplification of the dendritic arbor. Inhibiting NMDAR, LTCC, MCU or autophagy/mitophagy, or stimulating the activity of NCLX, each confers protection against excitatory dendritic mitochondrial toxicity.

In classic excitotoxicity, a transient episode of ischemia causes the extracellular concentrations of glutamate to rise. This results in widespread stimulation of both synaptic and extrasynaptic NMDARs, resulting in massive influx of sodium and calcium (**Figure 1A**). Ischemia induced neuronal damage is attenuated by pretreatment with an NMDAR antagonist, implicating glutamate toxicity (Simon et al., 1984). Apart from glutamate, earlier studies also implicated kainate and *N*-methyl-DL-aspartate in calcium dependent neuronal cell death (Berdichevsky et al., 1983). Whereas sodium may mediate the initial, reversible swelling of neurons, irreversible excitotoxic injury is believed to be mediated primarily by elevated calcium levels (Choi, 1995). The data suggest that transient elevations of intracellular calcium is tolerated by the cell and is reversible, whereas sustained calcium overload causes activation of intracellular enzymes (Choi, 1987) and a wave of mitochondrial collapse propagating to the cell body (Greenwood et al., 2007) to cause cell death. The initial glutamate stimulated calcium influx also triggers secondary increases in cytosolic calcium through other mechanisms, which are tightly correlated with neuronal cell death (Randall and Thayer, 1992). Classic excitotoxicity thus involves multiple calcium-dependent pathways initiated in the cytosolic compartment.

In addition to activating calcium-dependent degradative enzymes, such as calpains, phospholipases, and endonucleases, engagement of extrasynaptic NMDARs shuts off CREB signaling (Hardingham et al., 2002), while activating death associated protein kinase 1 (DAPK1) and neuronal nitric oxide synthase (nNOS) bound to the NR2B cytosolic tail (Tu et al., 2010; Martel et al., 2012). Calpain inhibitors confer dose-dependent protection from excitotoxic cell death, as well as preventing mitochondrial permeability transition and release of pro-death factors (Lankiewicz et al., 2000). In turn, inhibiting mitochondrial calcium uptake confers at least partial protection against cell death, depending on the severity of injury (Stout et al., 1998; Qiu et al., 2013), and mitochondrial permeability transition further amplifies calpain as well as caspase activation (Ferrand-Drake et al., 2003). However, adult neurons dying from ischemic/hypoxic injuries do not exhibit classic apoptotic morphology, most likely due to calpain-mediated inactivation of procaspase-9 (Volbracht et al., 2005). These data implicate m-calpain activation as a major factor in both mitochondrial and non-mitochondrial mechanisms of excitotoxic cell death.

## Calcium Dysregulation in Chronic Neurodegeneration

In recent years, it has become clear that calcium dysregulation also contributes to chronic neurodegeneration in relation to AD (Lopez et al., 2008; Anekonda and Quinn, 2011), ALS (Joo et al., 2007), and PD (Cali et al., 2014) and its related dementias: Parkinson disease with dementia (PDD) (Verma et al., 2017) and Dementia with Lewy Bodies (DLB) (Overk et al., 2014), which by convention are distinguished by the relative timing of cognitive and motor symptoms. A variety of clinical studies have converged on the possible neuroprotective role of calcium channel inhibitors for PD. In particular, both substantia nigra pars compacta neurons and cortical neurons express L-type voltage-gated channels (Guzman et al., 2009). Interestingly, L-type calcium channel inhibitors confer protection of SN and cortical neuron types in both toxic (Ilijic et al., 2011) and genetic (Cherra et al., 2013) models of PD. Moreover, use of centrally acting dihydropyridine L-type calcium channel blockers for hypertension treatment may reduce the risk of PD (Becker et al., 2008; Ritz et al., 2010). These studies emphasize the importance of understanding how neuronal calcium dysregulation contributes to structural and functional changes early in the neurodegenerative process.

In the remainder of this review, we summarize data that supports the concept of a new pathway of sublethal excitatory injury focused near sites of calcium entry, which contributes to dendrite retraction rather than propagating to the soma to cause cell death. We propose the term EMT, to emphasize the key role of mitochondrial calcium dysregulation in this pathway of neurodegeneration. Dysregulation of post-synaptic calcium handling may be triggered by several mechanisms involving proteins implicated in PD, ALS, or AD. The resultant elevations in cytosolic and mitochondrial calcium result in mitochondrial calcium injury, mitochondrial autophagy (mitophagy) and depletion of mitochondria from dendrites (**Figure 1B**). In contrast to excitotoxicity, which is predominantly triggered by excessive extracellular glutamate release, this pathway may also be triggered by post-synaptic changes in excitability, calcium buffering/recovery, and mitochondrial calcium influx or efflux.

## Calcium, Mitochondrial Content/Distribution, and Neuronal Arborization

Mitochondria play a key role in buffering and shaping cytosolic calcium transients, as well as a critical permissive role for neurite outgrowth, maintenance, and remodeling of axo-dendritic extensions. Before considering disease-linked alterations, basic mechanisms underlying these processes are briefly summarized below.

### Regulation of Mitochondrial Calcium Handling

Mitochondrial act to buffer intracellular calcium levels through high capacity, low affinity uptake by the mitochondrial calcium uniporter (MCU) complex (Baughman et al., 2011; De Stefani et al., 2011). As such, disease-associated changes in excitatory activity or other sources of increased cytosolic calcium will invariably affect mitochondria. Fine-tuning of MCU function is mediated by accessory proteins MICU1 (Perocchi et al., 2010), MICU2 (Plovanich et al., 2013), EMRE (Sancak et al., 2013), MCUR1 (Mallilankaraman et al., 2012), and MCUb (Raffaello et al., 2013). Mitochondrial calcium uptake is balanced by the activity of sodium/calcium antiporters, such as NCLX (Palty et al., 2010), which release calcium back into the cytosol. Another mitochondrial inner membrane protein LETM1 may act to mediate calcium uptake in response to moderate increases in cytosolic calcium as well as acting in calcium extrusion from the matrix (Doonan et al., 2014), although this latter effect is controversial (De Marchi et al., 2014). Changes to the numbers or function of ER-mitochondrial contact sites may also affect mitochondrial calcium homeostasis (Raffaello et al., 2016), and this process may be regulated by Parkin (Cali et al., 2013), whose mutations cause recessive PD.

From a physiological perspective, calcium uptake into the mitochondrial matrix results in enhanced respiratory function, tuning mitochondrial function to synaptic activity (Bianchi et al., 2004; Vos et al., 2010). However, with massive or sustained calcium stress, this response may result in mitochondrial injury from calcium overload. Following classic excitotoxic glutamate stimulation, excess mitochondrial calcium uptake results in ROS production (Reynolds and Hastings, 1995), collapse of membrane potential and opening of the permeability transition pore (Li et al., 2009), and induction of neuronal cell death (Stout et al., 1998). Indeed, MCU overexpression exacerbates NMDAR-mediated mitochondrial depolarization and excitotoxic cell death (Qiu et al., 2013). As discussed below, calcium uptake via MCU may also contribute to sublethal pathways of mitochondrial injury sufficient to trigger mitophagy and subsequent dendritic remodeling.

### Calcium, Mitochondria and Dendritic Remodeling

Mitochondria play a key role in the maintenance of dendritic integrity in neurons. Neurons are heavily dependent on the proper function and distribution of mitochondria to stay healthy (Gusdon and Chu, 2011). These accumulate or move toward regions of high energy demand, such as the growth cones of developing neurons (Morris and Hollenbeck, 1993) or regions of higher synaptic activity (Chang et al., 2006). The density and distribution of dendritic mitochondria regulates dendritic morphology, spinogenesis, and the plasticity of spines and synapses (Li et al., 2004). Moreover, in genetic models of neurodegeneration, loss of dendritic mitochondria precedes dendritic retraction (Cherra et al., 2013). This may relate to the requirement for sufficient mitochondrial densities not only to support synaptogenesis during development (Ishihara et al., 2009), but also for maintenance of dendritic arbors in mature neurons (Lopez-Domenech et al., 2016). Depletion of dendritic mitochondria may occur through reduced mitochondrial biogenesis, increased mitochondrial degradation, or alterations in mitochondrial transport.

Two important signals affect mitochondrial movement within a neuronal cell. (i) The energy status of the neuron modulates the transport of mitochondria, wherein high ATP levels increases mobility and high ADP concentration causes either slowing or total arrest of mitochondrial movement. Interestingly, mitochondrial velocity is also decreased in the close vicinity of a spine (Mironov, 2007). (ii) Changes in intracellular calcium levels regulate mitochondrial motility, wherein high cytosolic calcium levels decrease mitochondrial mobility. This may account for the tendency of mitochondria to accumulate near glutamate receptors, where they are situated to provide ATP and to buffer incoming intracellular calcium. Activity dependent mitochondrial movement was elegantly shown by Li et al. (2004), with the number of mitochondria in dendritic protrusions increased by repetitive KCl depolarization. Although the majority of mitochondria were present in the dendritic shaft, a small fraction of mitochondria was observed in the spine itself (Li et al., 2004). Interestingly, even under basal conditions, levels of mitochondria derived oxidative stress is higher in dendrites than in the soma, consistent with an increased bioenergetic demand associated with buffering calcium near a synapse (Dryanovski et al., 2013).

The stimulation of NMDA receptors leads to the activation of protein kinases, such as Ca^(2+)^/calmodulin-dependent protein kinase (Ojuka, 2004), AMP kinase, and mitogen activated protein kinases, such as ERK1/2 (Yun et al., 1999). Whereas AMP kinase (Ojuka, 2004) and ERK1/2 (Wang et al., 2014) show opposite effects on mitochondrial biogenesis, activation of either signaling pathway serves to promote autophagy or mitophagy (Pattingre et al., 2003; Meijer and Codogno, 2007; Dagda et al., 2008; Bootman et al., 2018). The ability of neuronal cells to undergo mitochondrial biogenesis regulates the outcome of mitophagy stimulation (Zhu et al., 2012). It is reasonable to surmise that an imbalance in the rates of mitochondrial degradation by mitophagy and replacement by biogenesis/transport will similarly determine the degree of mitochondrial depletion from dendrites.

## Sublethal Excitatory Calcium Dysregulation in Chronic Neurodegeneration

In contrast to classic excitotoxicity, in which massive, acute elevations in glutamatergic neurotransmission results in both non-mitochondrial and mitochondrial pathways of cell death, functional impairment and shrinkage of the synaptic-dendritic arbor likely occur long before cell death in chronic neurodegenerative diseases. Interestingly, sublethal stimulation of NMDA receptors decreases dendrite outgrowth in immature neurons (Monnerie et al., 2003), but the impact on dendritic integrity in mature neurons is less understood. Nevertheless, there are a growing number of studies implicating chronic elevations in excitatory post-synaptic potentials and cytosolic calcium in models of neurodegenerative diseases, which may be due to either pre-synaptic or post-synaptic changes.

### Parkinson’s Disease

The movement symptoms that characterize PD result from degeneration of dopaminergic substantia nigra neurons in the midbrain, which project to the striatum. In addition, PD patients frequently experience olfactory and autonomic dysfunction, mood disorders, and cognitive/executive dysfunction. In addition to playing a key role in cortical neuron function, glutamate plays an important role in modulating dopaminergic neurotransmission, acting on both pre-synaptic and post-synaptic sides. Dopaminergic midbrain neurons express both synaptic and extrasynaptic glutamate receptors (Wild et al., 2015) and are susceptible to classic NMDA excitotoxicity (Kikuchi and Kim, 1993). Excitatory cortical input also modulates striatal neurotransmission in both direct and indirect basal ganglia pathways (Stocco, 2012). While dementia may represent a late stage development in some forms of PD, early cognitive-executive dysfunction represents the defining feature of DLB as well as in several forms of familial PD.

Mutations in the *LRRK2* gene, which encodes leucine-rich repeat kinase 2, represent the most frequent known cause of PD (Gandhi P.N. et al., 2009). Recent studies using cultured primary neurons transfected with disease-linked G2019S and R1441C mutations of *LRRK2* implicate increased excitatory neurotransmission as one of the earliest pathogenic changes, preceding subsequent dendritic degeneration (Plowey et al., 2014). EPSP frequency was elevated basally, and neurons showed enhanced responses to NMDA and AMPA. Interestingly, memantine, a partial NMDA antagonist conferred protection against subsequent dendritic simplification and loss, implicating an excitatory pathogenesis (Plowey et al., 2014). Increased post-synaptic excitatory neurotransmission was also observed in hippocampal slice cultures of LRRK2-G2019S transgenic mice (Sweet et al., 2015). Interestingly, LRRK2-G2019S knockin mice exhibit an early stage of hyperactivity, accompanied by increased striatal glutamate and dopamine neurotransmission (Volta et al., 2017). Thus, primary neuron cultures, slice cultures and *in vivo* studies all support an early role for increased excitatory synaptic activity in several mutant LRRK2 models.

Other mechanisms may also contribute to increased intracellular calcium in mutant LRRK2-expressing neurons. As mentioned above, L-type voltage-gated channels contribute to Ca^2+^ influx during an action potential. Expression of either the G2019S or the R1441C mutation in LRRK2 dysregulates intracellular calcium homeostasis in response to KCl depolarization (Cherra et al., 2013). Calcium chelators or inhibitors of L-type calcium channels confer protection in this system. Calcium release from lysosomal stores has also been implicated in mutant LRRK2 pathogenesis (Gomez-Suaga et al., 2012; Hockey et al., 2015).

Oligomeric α-synuclein, implicated in both dominant familial and sporadic PD/DLB, elicits increased cytosolic calcium uptake through effects on AMPARs (Huls et al., 2011). This creates increased susceptibility to MPP+ toxicity (Lieberman et al., 2017). Interestingly, α-synuclein oligomers can act to increase intracellular calcium levels by forming pores in the plasma membrane (Pacheco et al., 2015). Furthermore, the neurite retraction and increased intracellular calcium elicited by the A53T mutation in α-synuclein are exacerbated by concurrent expression of PINK1-W437X (Marongiu et al., 2009), implicating a mechanistic convergence between dominant and recessive forms of PD. It has been proposed that the reduced mitochondrial membrane potential often observed in PINK1 knockdown/knockout cells (Exner et al., 2007; Dagda et al., 2011, 2014; Huang et al., 2017) may serve to limit mitochondrial calcium uptake, exacerbating excitotoxic injury (Heeman et al., 2011). When post-synaptically expressed, Parkin participates in pruning excitatory synapses (Helton et al., 2008). This may represent another point of convergence between dominant and recessive PD, as either loss of Parkin function or dominant LRRK2 mutations would tend to increase excitatory synapses, conferring enhanced vulnerability to excitatory injury. Taken together, dysregulated neuronal calcium handling resulting in increased cytosolic levels forms a common theme in multiple forms of familial Parkinsonism.

### Alzheimer’s Disease

Alzheimer’s disease is the most common age-related neurodegenerative disease, characterized by memory deficits and the pathological hallmarks of neuritic plaques and neurofibrillary tangles. Proteins that are pathologically implicated in AD include the amyloid beta peptides (Aβ) and the microtubule associated protein tau. Calcium mishandling has been implicated in AD and elevated serum calcium levels are well correlated with cognitive decline in aging (Lopez et al., 2008; Popugaeva et al., 2017). Oligomeric Aβ, proteolytic products of the amyloid precursor protein (APP) that is mutated in familial AD (fAD), are enriched in the plaques that typify the disease and are primary culprits for initiating this calcium dysregulation. Neurons exposed to Aβ oligomers elicit elevations in somatic, dendritic, and synaptic calcium in neurons (Arbel-Ornath et al., 2017; Zhao et al., 2017) and contribute to excitotoxic neuron death (Mattson et al., 1992; Shankar et al., 2008). In mice engineered to co-express fAD mutations in APP_swe_ and Presenilin 1(PS1_G384A_), a gamma secretase that cleaves APP to generate Aβ peptides, hyperactive neurons are observed in the hippocampus and cortex of young animals, prior to formation of plaques (Busche et al., 2012). Mutations in presenilins alone have also been reported to elicit endoplasmic reticulum calcium overload, with post-translational modification of neuronal ryanodine receptors further promoting calcium leakage into the cytosol (Lacampagne et al., 2017; Popugaeva et al., 2017). Emerging theories suggest long-term Aβ-dependent calcium dysregulation may trigger a cascade of deficits in homeostatic machinery that result in loss in neural network activity (Frere and Slutsky, 2018). For example, elevated calcium plays a key role in promoting tau pathology (Zempel et al., 2010), a component of neurofibrillary tangles that characterize an intermediate step in AD progression, through activation of numerous kinases thought to mediate tau’s effects (Mairet-Coello et al., 2013).

### ALS-FTD

Amyotrophic lateral sclerosis is a debilitating disorder affecting upper and lower motor neurons in the cortex, brainstem, and spinal cord (Rowland and Shneider, 2001). Similar to other neurodegenerative diseases, most of the ALS cases are sporadic and 10% of the cases are familial. There is both genetic and pathological overlap between ALS and forms of FTD characterized by accumulations of TAR DNA binding protein-43 (TDP-43) and/or with mutations in C90rf72 (Ling et al., 2013). Motor neurons in ALS are vulnerable to excitotoxic injury as these neurons highly express AMPAR calcium channels (Williams et al., 1997; Corona and Tapia, 2007), accompanied by low expression of calcium buffering proteins (Alexianu et al., 1994; Jaiswal, 2013). In addition, mitochondrial dysfunctions have been reported in post-mortem brain tissues of ALS patients (Sasaki and Iwata, 1996; Kong and Xu, 1998) as well as in animal models of ALS (Nguyen et al., 2009; Santa-Cruz et al., 2016). Given the susceptibility of these neurons to calcium overload induced toxicity, mitochondria play important calcium buffering roles in these neurons (Smith et al., 2017). Excessive exposure to glutamate can lead to glutamate induced excitotoxicity (Stout et al., 1998). Increased glutamate toxicity could be due to enhanced synaptic activity (Milanese et al., 2011) or dysfunctional reuptake by neighboring glial cells (Fray et al., 1998), which can cause persistent activation of AMPAR and increased cytosolic calcium burden leading to mitochondrial calcium overload (Goodall and Morrison, 2006). Interestingly, increased excitatory activity and dendritic spine numbers are observed in early pre-symptomatic stages of the TDP-43(Q331K) model of ALS (Fogarty et al., 2016). Thus, genetic mouse models of all three diseases, PD, AD, and ALS indicate an early phase of excitatory hyperactivity.

## Excitatory Mitochondrial Toxicity (EMT) in Chronic Neurodegenerative Diseases

In this section, we discuss how PD-linked changes in mitochondrial calcium transport proteins act in concert with sublethal elevations in excitatory neurotransmission to elicit mitochondrial injury and mitochondrial depletion from dendrites. Mitochondrial depletion then contributes to retraction and simplification of the dendritic arbor. In contrast to excitotoxicity, which rapidly results in the classic red, dead neuron observed in stroke, calcium injury triggered autophagy/mitophagy plays a key role in dendritic simplification observed in several models of PD. Given that dendritic pathology is observed in post-mortem studies of PD (Patt et al., 1991), AD (Brizzee, 1987), and ALS (Genc et al., 2017), the review closes with a discussion of the potential implications of the EMT mechanism for sporadic PD and other neurodegenerative diseases.

### EMT in the LRRK2 Model

Shrinkage of the dendritic arbor represents one of the most frequently reported phenotypes exhibited by neurons expressing disease-linked mutations in LRRK2 (MacLeod et al., 2006; Ramonet et al., 2011; Winner et al., 2011; Cherra et al., 2013; Reinhardt et al., 2013; Plowey et al., 2014; Verma et al., 2017). This may be related to effects on microtubule dynamics, endosomal trafficking and/or autophagy [Reviewed in Ref. (Verma et al., 2014)]. Among the earliest changes exhibited by primary cortical neurons transfected with LRRK2-G2019S or LRRK2-R1441C are increased activity-dependent calcium influx through glutamate receptors and L-type calcium channels (Cherra et al., 2013; Plowey et al., 2014). This is followed by loss of mitochondria specifically from the dendritic compartment, which precedes subsequent neuritic retraction (Cherra et al., 2013). The loss of mitochondria can be blocked by inhibiting autophagy or expressing a phosphomimicking mutation of the autophagy protein LC3 (Cherra et al., 2013), which is predicted to impair the cardiolipin pathway of mitophagy (Chu et al., 2014). Mitochondrial fission is often required for efficient mitophagy (Twig et al., 2008; Dagda et al., 2009). Interestingly, mutant LRRK2 regulates Drp1-dependent mitochondrial fission as well as activating ULK1 to mediate mitophagy (Zhu et al., 2012; Su and Qi, 2013). The mechanism that leads to mitophagy of dendritic mitochondria downstream of mutant LRRK2-induced cytosolic calcium uptake was recently delineated using primary neurons transfected with genetically encoded calcium sensors (Verma et al., 2017). As expected, LRRK2-G2019S and -R1441C increased intracellular calcium uptake in response to stimulation, and this was accompanied by increased mitochondrial calcium uptake in dendrites. The increased dendritic mitochondrial calcium uptake persisted even in permeabilized neurons exposed to the same calcium concentrations, implicating increased mitochondrial calcium transport capacity in dendrites of mutant-LRRK2 expressing neurons. Further investigation revealed that mutant LRRK2-transfected neurons, as well as fibroblasts from PD patients with the G2019S and R1441C mutations, showed increased mRNA and protein expression of MCU and MICU1, with no changes in MICU2 or NCLX expression (Verma et al., 2017). Neurons treated with MCU inhibitors exhibited decreased mitophagy and were protected from dendritic simplification induced by mutant LRRK2. These data implicate calcium-dependent injury to mitochondria within dendrites, and their subsequent mitophagic elimination, as mechanisms linking increased excitatory input with dendritic simplification.

### EMT in the PINK1 Model

The recessive PD-linked gene PINK1, which is targeted to mitochondria via a classic N-terminal mitochondrial targeting sequence, has also been implicated in regulation of mitochondrial calcium homeostasis. As mentioned above, decreases in mitochondrial membrane potential are likely to have multiple consequences including decreased cytosolic calcium buffering and the loss of mitochondria due to mitophagy. Indeed, PINK1-deficient systems exhibit impaired calcium recovery (Heeman et al., 2011) and elevated basal mitophagy in neuronal cells (Dagda et al., 2009; Chu, 2010) and in pancreatic beta cells *in vivo* (McWilliams et al., 2018), evidently through one of several PINK1- and Parkin-independent mechanisms (Chu et al., 2013; Strappazzon et al., 2015; Bhujabal et al., 2017). In particular, mitochondrial calcium overload has been implicated, as inhibiting mitochondrial calcium uptake, in cells co-expressing α-syn A53T and Pink1 W437X, restores Δ*Ψ*_m_ and rescues neurite outgrowth (Marongiu et al., 2009).

Like primary neurons expressing mutant LRRK2, neurons cultured from PINK1 knockout mice to model recessive PD pathogenesis exhibit reduced dendritic arbors (Dagda et al., 2014). Interestingly, PINK1 was shown to regulate calcium efflux via NCLX, with PINK1 deficiency causing mitochondrial calcium overload (Gandhi S. et al., 2009). Indeed, it has recently been shown that PINK1 promotes PKA-dependent phosphorylation and activation of NCLX (Kostic et al., 2015). PINK1-deficient neurons are susceptible to dopamine toxicity, and expression of the NCLX-S258D phosphomimic mutant restores mitochondrial calcium efflux and confers neuroprotection. LETM1, another mitochondrial calcium transporter, represents a direct phosphorylation target of PINK1 (Huang et al., 2017). As LETM1 mediates both calcium influx and efflux (Doonan et al., 2014), the effects of LETM1 activation or loss of function are difficult to predict. Nevertheless, impaired mitochondrial calcium efflux, in a parallel pathway to the effects of PINK1 deficiency on NCLX activity, appears to represent the key pathogenic factor.

### Converging Mechanisms in Neuroprotection

Whereas episodic calcium entry into the mitochondrial matrix stimulates respiration to adjust mitochondrial output to bioenergetic needs, chronically elevated cytosolic calcium oscillations elicit mitochondrially derived ROS, elevated mitophagy and decreased basal mitochondrial content in dopaminergic substantia nigra neurons (Liang et al., 2007; Guzman et al., 2018). While this occurs under normal conditions for pacemaking cells, such as substantia nigra neurons (Guzman et al., 2018), the mitochondrial response to cytosolic calcium influx is exaggerated in disease states, triggering mitophagy and loss of dendritic mitochondria, followed by a delayed degeneration of dendritic processes (Cherra et al., 2013; Verma et al., 2017). Inhibiting cytosolic calcium influx through NMDA receptors (Plowey et al., 2014) or L-type calcium channels (Cherra et al., 2013; Guzman et al., 2018) prevents the elevated dendritic mitophagy and restores mitochondrial density and dendrite lengths. Inhibiting mitochondrial calcium uptake via MCU confers complete restoration of dendrite lengths (Verma et al., 2017), supporting a central role for mitochondrial calcium mishandling in EMT.

It is likely that increased mitochondrial calcium uptake in the mutant LRRK2 model represents, at least initially, a compensatory response to increased excitatory input. Indeed, MCU and MICU1 are transcriptionally upregulated in mutant LRRK2-expressing neurons and fibroblasts through activation of the ERK1/2 signaling pathway (Verma et al., 2017), which has been proposed to mediate several effects of mutant LRRK2 (Carballo-Carbajal et al., 2010; Reinhardt et al., 2013). Interestingly, a similar elevation in phospho-ERK2, MCU, and MICU1 expression is observed in cortical brain samples from patients with sporadic PD/PDD (Verma et al., 2017, suggesting that enhanced susceptibility to mitochondrial calcium overload could contribute to sporadic disease as well.

One of the models of sporadic disease involves complex I inhibitors, such as MPP+ and rotenone, as PD patients exhibit systemically decreased complex I activity (Greenamyre et al., 2001). Mitochondrial calcium overload has been implicated specifically in the substantia nigra, but not the relatively resistant ventral tegmental area, in the MPP+ model of parkinsonian complex I deficiency (Lieberman et al., 2017). Downregulating autophagy through PKA-mediated phosphorylation of LC3 confers protection against neurite retraction in this model (Cherra et al., 2010), although the potential protective role of modulating MCU or NCLX activities remains unexplored.

While stimulated mitochondrial calcium uptake is either unchanged (Kostic et al., 2015) or slightly decreased (Huang et al., 2017) in PINK1-deficient cells, future studies are needed to determine whether or not there may be concurrent disruption of mitochondrial efflux mechanisms in the mutant LRRK2 model. Irregardless, mutant forms of NCLX that mimic phosphorylation at the PINK1/PKA-regulated NCLX-S258 site (Kostic et al., 2015) confer protection from mutant LRRK2-mediated dendritic simplification to the same extent as inhibiting MCU (Verma et al., 2017). Likewise, inhibition of MCU is neuroprotective in a zebrafish model of PINK1 deficiency (Soman et al., 2017), indicating that reducing the likelihood of mitochondrial calcium overload through either the influx or efflux pathways may be effective irregardless of the original predisposing mechanism.

### Implications for Other Diseases

It is known that inhibiting mitochondrial calcium uptake via MCU is beneficial in protecting against neuronal cell death after stroke (Abramov and Duchen, 2008) or during NMDA induced excitotoxic neuronal cell death (Qiu et al., 2013). Recent studies have also shown neuroprotective effects of inhibiting of MCU on Aβ induced microglial cell death (Xie et al., 2017), loss of hippocampal neurons in pilocarpine induced status epilepticus (Wang et al., 2015) or ischemia/reperfusion injury (Zhao et al., 2013). Interestingly, inhibition of MCU protects against neuronal ischemia-reperfusion injury by inhibiting excess mitophagy (Yu et al., 2016), similar the mechanism described in the mutant LRRK2 model (Verma et al., 2017). However, the potential role of sublethal mitochondrial injuries in triggering EMT has been much less studied outside of PD.

In particular, it is unknown if changes in MCU or NCLX expression or post-translational modifications may contribute to sensitivity to excitatory injury in these other diseases. It would be important to delineate whether or not the activities of kinases that regulate mitochondrial calcium transporters are altered in susceptible neurons. In addition to being elevated in patient brains with familial LRRK2 mutation (Verma et al., 2017), elevated ERK1/2 is also observed in sporadic PD/PDD/DLB (Zhu et al., 2002; Verma et al., 2017), AD (Perry et al., 1999), hypoxia-ischemia (Wang et al., 2003), and in organotypic spinal cord culture models of ALS-related TDP-43 pathology (Ayala et al., 2011). As ERK1/2 drives the changes in MCU and MICU expression observed in familial PD patient cells and models, it is possible that EMT contributes to dendritic retraction and simplification in a spectrum of neurodegenerative conditions.

While experiments involving the inhibition of autophagy support the conclusion that mitophagy contributes to mitochondrial depletion from dendrites, another key question to be considered is to understand why aging or diseased neurons fail to replace the degraded mitochondria. These factors may include age- or disease-related decline in mitochondrial biogenesis as observed in PD (Zheng et al., 2010; Zhu et al., 2012) or alterations in mitochondrial transport in neuronal processes as implicated in AD (Calkins and Reddy, 2011) and ALS (Magrane et al., 2012). Mitochondrial depletion would persist only if mitophagy is not balanced by mechanisms to replace the degraded mitochondria. It is thus conceivable that therapies targeting mitochondrial biogenesis or transport may also rescue the ill effects of dendritic EMT.

## Conclusion

Dendritic simplification is observed in mutant *LRRK2*-expressing neurons, in *PINK1* knockout neurons, in post-mortem sporadic PD patient neurons and in other neurodegenerative and neuropsychiatric conditions. We propose that dominant, recessive and sporadic contributions to altered mitochondrial calcium homeostasis converge on a process of EMT to mediate degeneration of the dendritic arbor observed in many neurodegenerative diseases. This may result from effects on the MCU complex itself, on mitochondrial calcium extrusion mechanisms and/or any change that result in greater cytosolic calcium levels. While an alteration in mitochondrial calcium handling on its own may be insufficient to cause neuronal injury, when combined with increased post-synaptic calcium fluxes that accompany excitatory synaptic activity, EMT leads to dendritic shortening and simplification by triggering unbalanced mitophagy and perisynaptic mitochondrial depletion, mechanisms that are distinct from classic excitotoxicity. Moreover, while dominant and recessive contributions to dendritic EMT occur through different mechanisms, interventions that either reduce mitochondrial calcium uptake via MCU or that target NLCX to enhance mitochondrial calcium release are reciprocally effective in both systems (Kostic et al., 2015; Soman et al., 2017; Verma et al., 2017). Future studies to determine whether increased excitatory activity observed in AD and ALS-FTD is also linked to dendritic simplification and spine loss via mitochondrial calcium overload will help determine whether or not therapies targeting EMT may have even broader applicability.

## Author Contributions

MV reviewed the literature, designed figures, and wrote sections of the manuscript. ZW contributed to concept development and edited the manuscript. CC developed the conceptual framework, wrote, and edited the manuscript.

## Conflict of Interest Statement

The authors declare that the research was conducted in the absence of any commercial or financial relationships that could be construed as a potential conflict of interest.
